# Antibodies and Antibody Derivatives: New Partners in HIV Eradication Strategies

**DOI:** 10.3389/fimmu.2018.02429

**Published:** 2018-10-23

**Authors:** Jorge Carrillo, Bonaventura Clotet, Julià Blanco

**Affiliations:** ^1^IrsiCaixa AIDS Research Institute, Institut de Recerca Germans Trias i Pujol, Badalona, Spain; ^2^Chair in AIDS and Related Illnesses, Centre for Health and Social Care Research (CEES), Faculty of Medicine, Universitat de Vic - Universitat Central de Catalunya, Vic, Spain

**Keywords:** broadly neutralizing antibodies, HIV persistence, effector functions, HIV reservoir, NK cells, ADCC

## Abstract

Promptly after primoinfection, HIV generates a pool of infected cells carrying transcriptionally silent integrated proviral DNA, the HIV-1 reservoir. These cells are not cleared by combined antiretroviral therapy (cART), and persist lifelong in treated HIV-infected individuals. Defining clinical strategies to eradicate the HIV reservoir and cure HIV-infected individuals is a major research field that requires a deep understanding of the mechanisms of seeding, maintenance and destruction of latently infected cells. Although CTL responses have been classically associated with the control of HIV replication, and hence with the size of HIV reservoir, broadly neutralizing antibodies (bNAbs) have emerged as new players in HIV cure strategies. Several reasons support this potential role: (i) over the last years a number of bNAbs with high potency and ability to cope with the extreme variability of HIV have been identified; (ii) antibodies not only block HIV replication but mediate effector functions that may contribute to the removal of infected cells and to boost immune responses against HIV; (iii) a series of new technologies have allowed for the *in vitro* design of improved antibodies with increased antiviral and effector functions. Recent studies in non-human primate models and in HIV-infected individuals have shown that treatment with recombinant bNAbs isolated from HIV-infected individuals is safe and may have a beneficial effect both on the seeding of the HIV reservoir and on the inhibition of HIV replication. These promising data and the development of antibody technology have paved the way for treating HIV infection with engineered monoclonal antibodies with high potency of neutralization, wide coverage of HIV diversity, extended plasma half-life *in vivo* and improved effector functions. The exciting effects of these newly designed antibodies *in vivo*, either alone or in combination with other cure strategies (latency reversing agents or therapeutic vaccines), open a new hope in HIV eradication.

## HIV persistence: the success and the failure or antiretroviral therapy

HIV, as other retroviruses, requires integration of the proviral genome into host cells to allow for transcription of viral genes and completion of the viral life cycle (1). Both events, integration and further transcription of integrated proviral genomes are strongly dependent on the activation status of target CD4^+^ T cells. Highly activated cells efficiently integrate and replicate HIV, while resting cells hardly support viral integration and show low or null transcriptional activity (2). Therefore, upon infection, a pool of latently infected cells bearing silent HIV proviral sequences, the HIV reservoir, is formed either by the ability of HIV to overcome integration restrictions in resting cells (3) or by the contraction of immune responses that allows some HIV-infected activated cells to return to a resting status, silencing viral transcription (4).

Current treatment of HIV infection, the combination antiretroviral therapy (cART), is mostly based on an array of inhibitors of several viral enzymes (reverse transcriptase, protease or integrase) and is extremely effective at blocking HIV replication, leading to a sustained suppression of plasma viremia at least below the limit of detection of standard assays (5). However, the persistence of the HIV reservoir and its spontaneous activation rapidly resume viral replication after treatment interruption. Recent data describing treatment interruption of very early treated individuals (Fiebig I) shows that the HIV reservoir is rapidly established after primoinfection (6). Although the HIV reservoir is relatively small in size, ranging from 1 to 100 latently infected cells per million of CD4^+^ T cells, cells, it may encompass long-lived cells, such as resting memory CD4^+^ T cells or macrophages (7, 8), The main consequence of this fact is that the pool of latently infected cells in treated HIV-infected individuals shows a slow decay overtime. The half-life of the HIV reservoir has been calculated in 3.75 years and therefore its natural eradication would require 60 years of continuous cART treatment (2). Several attempts to accelerate reservoir decay by combining new and more potent drugs in optimal cART regimens have shown some impact on viral and immune dynamics but failed to show a positive effect on the HIV reservoir decay rate (9–13). Consequently, HIV-infected individuals have to manage life-long coexistence with HIV, with therapy, and with their associated complications, such as chronic immune activation and inflammation, and drug toxicities. Ultimately, these effects may result in accelerated immunosenescence and aging (14) that associates with higher incidence of co-morbidities and mortality compared to non-infected individuals (8). Therefore, there is an emergent interest in developing safe and affordable curative strategies that would eliminate the need of lifelong therapy in HIV-infected individuals, either by completely eradicating the viral reservoir (sterilizing cure) or by empowering the immune control of HIV-replication (functional cure).

## Dynamics of HIV reservoir under cART: clues for cure

**Figure 1:**
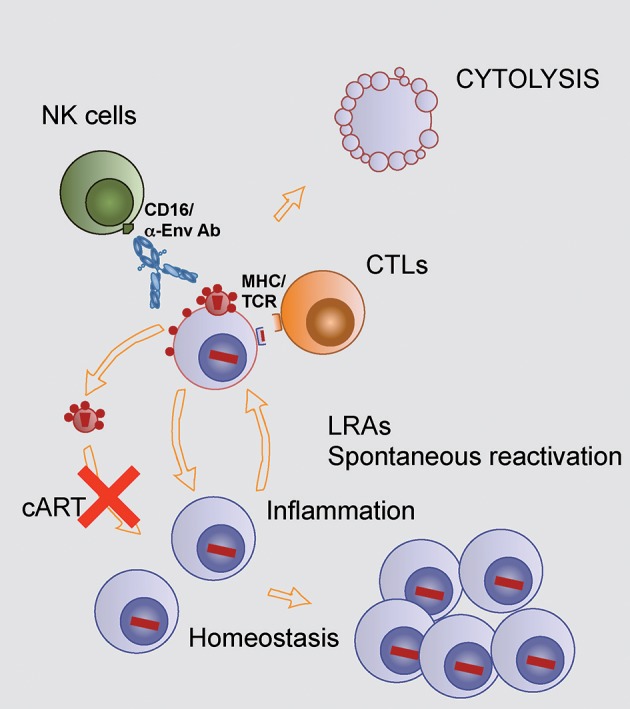
Mechanisms controlling HIV reservoir in treated HIV-infected individuals. The HIV reservoir, the pool of HIV latently infected cells, is drained by continuous stochastic activation leading to transient or stable transcription of HIV provirus. Transient reactivation may allow a return to latent infection, while stable reactivation will allow for presentation of viral peptides to HIV specific CD8 T cells, exposure of HIV Env on the surface of cells allowing NK mediated ADCC. Both mechanisms will lead to the lysis of infected cells. Reinfection of target cells by virions produced by reactivated cells is blocked by cART. On the other hand, stimuli that promote cell proliferation in the absence of viral transcription will increase the size of the reservoir (bottom right).

The complete eradication of the pool of latently infected cells in HIV-infected individuals has been approached from molecular, pharmacological and immune perspectives. Molecular approaches are based on sequence specific CRISPR-CAS-based tools that may allow for the excision or reactivation of proviral DNA from latently infected cells; however, their clinical efficacy and potential off-target effects are not yet known (15). In contrast, more classical pharmacological and immunological strategies have reached human trials and have been tested in HIV-infected individuals. All these approaches are designed to perturb the long-lived HIV reservoir (Figure 1). Although the pool of latently infected cells is stable overtime, it is not static. On the one hand, homeostatic control of T cells and inflammatory environment may induce the proliferation of latently infected cells (16, 17). When this proliferation occurs in the absence of HIV transcription, it results in an increase in the HIV reservoir size, this is the case of HIV-infected individuals treated with IL-7 (18). Furthermore, clonal expansion of latently infected cells driven by antigen specificity or specific integration sites has also been described in HIV-infected individuals (19, 20).

On the other hand, HIV transcription in latently infected cells can become spontaneously and stochastically activated by different stimuli, also including specific antigens or nonspecific inflammatory signals, that reverse epigenetic silencing of HIV transcription (21). Once reactivated, cells will produce viral particles whose infectivity will be blocked by cART (Figure 1), and will expose viral antigens, both mature envelope glycoproteins (Env) on the cell surface and viral peptides presented by MHC will allow the immune system to recognize the reactivated cell before a potential return to a resting status or cytolysis mediated by cytopathic effects of HIV proteins (Figure 1). Immune recognition will accelerate the lytic process either by HIV-specific CD8+ cytotoxic T cells (CTL) or by CD16+ NK cells sensing antibodies bound to Env (Figure 1). However, the extent of both CTL and NK mediated cell lysis in HIV-infected individuals could be incomplete. In the case of CTL, a broad and properly stimulated response seems to be required to overcome the dominance of escape mutations and the inherent resistance of reactivated cells to CTL mediated killing (22–24). Regarding antibodies, the main limitation is the lack of good-quality antibodies against HIV Env and the potential perturbation of both B and NK cell functions induced by HIV infection (25, 26). Despite these limitations, indirect evidence link both CTL and NK cell function to the reservoir size in HIV-infected individuals (27–29).

Irrespective of the cytolytic mechanisms, the combination of reactivation (kick) and lysis (kill) will result in a continuous reduction of the HIV reservoir size. Therefore, kick-and-kill strategies are paradigmatic in the purge of the HIV reservoir (30). Optimization of kick is exploring a wide range of latency reversing agents (LRA). Several compounds acting at different levels of the control of HIV transcription, including histone deacetylase (HDAC) inhibitors and Toll-like receptors (TLR) agonists, have been tested in *in vitro* assays or in *in vivo* animal models to screen them for potential use in humans (31). Certainly, some of them have reached clinical trials in HIV-infected individuals, such as the HDAC inhibitors valproic acid, disulfiram, vorinostat, panobinostat, or romidepsin and the protein kinase C modulator briostatin (32–38). These trials yielded, at best, promising results in terms of HIV reactivation, showing transient increases in cell associated HIV RNA levels; however, no changes in HIV reservoir size were observed. Taken together, these data suggest that a better killing step is necessary to impact on the reservoir size. Thus, enabling the immune system to rapidly kill kicked cells seems to be a necessary step in cure strategies. Although, CTL based strategies, namely therapeutic vaccination aimed at inducing new CTL specificities, is an active field (39); antibody-based therapies have emerged as a new and powerful tool (40). Some reasons that explain the renewed interest in antibodies are the isolation of broad and highly potent anti-HIV antibodies, the demonstration of their safety and antiviral activity *in vivo* and the idea that antibodies display immunomodulatory activities beyond the antiviral activity.

## Antibodies, antiviral agents beyond ART

**Figure 2:**
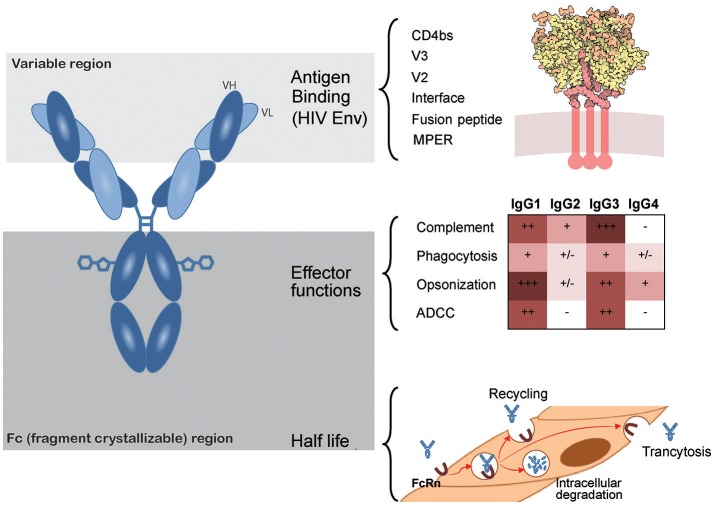
Main antibody features. Antibodies are glycosylated heterodimeric molecules showing a variable region in both light and heavy chains that determine antigen binding. For bNAbs, antigen binding is located on the indicated vulnerability sites of the HIV Env glycoprotein formed by heterotrimers of gp120 (yellow) and gp41 (red in the upper right panel). The crystallizable fragment of the antibodies (Fc) encompasses all constant regions and is responsible for the effector functions. Different antibody subtypes show selective effector functions (color coded displayed in the middle right panel). Furthermore, the Fc region also regulates plasma half-life of antibodies, as they are continuously recycled, degraded or transcytosed by endothelial cells through neonatal Fc receptors (FcRn, lower right panel). HIV Env picture (http://www.rcsb.org/pdb/101/motm.do?momID=169) is from David S. Goodsell and the RCSB PDB under Creative Commons.

Antibodies share with cART the capacity to block HIV replication, in the case of antibodies by their ability to inhibit HIV entry, also known as neutralizing activity. Direct antiviral or neutralizing activity depends on the variable region of the antibody that is defined by the N-terminal domains of the heavy and light chains of the molecule (Figure 2). The HIV Env is the sole viral protein exposed on virions and productively infected cells and is therefore the target for HIV neutralizing antibodies (41). Env is a heterotrimer of gp120 and gp41 subunits, with a high structural complexity, sequence variation and plasticity (42, 43). Despite this, several potent and broad neutralizing antibodies (bNAbs) have been identified that bind to relatively conserved and functionally relevant regions of Env. These epitopes, called vulnerability sites, are the CD4 binding site, the external V2 or the V3 loops in gp120, the gp120/gp41 interface and the fusion peptide or the membrane proximal external region (MPER) of gp41 (Figure 2) (42, 44, 45).

Unlike cART, antibodies can be considered polyfunctional molecules as they can mediate several antiviral functions combining direct blockade of viral infectivity (neutralization) and indirect immunological mechanisms (effector functions) that require the recruitment and activation of immune cells, such as NK cells or macrophages. Effector functions depend on the interaction of the fragment crystallizable (Fc) region of antibodies and specific Fc-Receptors (FcRs) differentially expressed on the surface of immune cells. The Fc domain is defined by the heavy chain of antibodies that also defines the isotype (46). For the main antibody isotype, IgG, four subtypes have been described, among them IgG1 and IgG3 are the most active mediating effector functions in viral infections (47). In this way, IgG1 and IgG3 can act as a “linker,” facilitating the interaction between immune-effector cells and virions or infected cells and promoting their elimination (Figure 2). Among the functions assigned to the Fc portion of antibodies, complement-mediated lysis, antibody-dependent cellular cytotoxicity (ADCC) and Antibody-dependent phagocytosis (ADCP) are highlighted because they are highly effective in killing and removing infected cells, thereby contributing to antiviral functions. Importantly, interactions of antigen bound antibodies with immune cells also act as a danger signal and are part of the communication network of the immune system to improve immune responses at the local or systemic level. One of the most relevant examples is the activation of conventional and plasmacitoid dendritic cells (DC) by immunocomplexes (IC). Such IC recognition leads to enhanced antigen uptake and presentation, allowing induction of stronger humoral and cellular antiviral immune responses, reviewed in (48).

Although Fc-mediated effector functions need for the antibody to recognize the antigen on the surface of infected cells, they are independent of the neutralizing potential. Therefore, both neutralizing and non-neutralizing antibodies could be effective mediating Fc-dependent antiviral functions. The best example is provided by the RV144 trial (49). In this trial involving more than 1,6000 individuals, a combination of Env immunogens failed to elicit a strong cross-neutralizing humoral response, but induced antibodies with ADCC activity that correlated with protection in individuals showing low IgA response (50, 51). Interestingly, Hessell et al. showed that the protective role of the IgG1b12 against SHIV acquisition observed in non-human primate (NHP) models was associated with the Fc-FcR interaction but not with complement activation (52). Therefore, these results suggested that the neutralizing capacity of the antibody is not enough to confer protection against HIV and probably the combination of both neutralization and Fc-dependent effector function, such as ADCC or ADCP are required. Accordingly, bNAbs whose Fc portion was modified to increase binding to activating FcR showed an improved protective capacity when they were assayed in humanized mice models (53).

In addition to the effector functions described above, the Fc-portion of IgGs is also responsible for the interaction with the neonatal Fc receptor (FcRn) widely expressed on endothelial cells. The interaction with this receptor plays a major role in the control of the plasma half-life of antibodies by regulating the acidic degradation of IgGs into the lysosome of endothelial cells (54). The mechanism is based on the pH dependence of the binding of antibodies to FcRn. Binding shows higher affinity at pH 6, which is the endosomal pH, and low affinity at neutral pH (extracellular). This fact enables antibodies taken up by endothelial cells to remain bound to the FcRn and recycle to the cell surface, avoiding the acidic degradation. Once the antibody/FcRn complex reaches the extracellular neutral pH compartment, antibodies are released. Importantly, besides regulating plasma half-life, this recycling process also modulates antibody transcytosis to tissues (55). Taking advantage of this mechanism, a modified VRC01 antibody (VRC01-LS), which showed an improved binding to the FcRn at pH = 6, provided superior protective capacity than the wild type version in rhesus macaques, not only by increasing its *in vivo* half-life but also by reaching a higher concentration at mucosal level (56).

Indirectly, antibodies might also contribute to improve the CTL response against HIV-1. The treatment of rhesus macaques with a combination of 3BNC117 and 10-1074 antibodies very early after SHIV infection, helps to control the infection by a mechanism in which CD8+T cells seem to be fundamental (57). Nevertheless, it is possible that antibodies contribute to promote the T cell response by improving the antigen presentation capacity of DCs. As mentioned above, IC composed of antibodies, virions and probably complement, can be efficiently captured by DCs, via FcR, processed and presented to T cells, improving the cellular response against viral antigens (48). This process seems to be highly dependent on timing, since this effect has not been observed when antibodies were administered before infection or during the chronic phase, and, probably might also depend on the neutralizing capacity and isotype of the antibody used.

In summary, antibodies show a wide range of antiviral activities that make them highly attractive as anti-HIV-1 agents. They can reduce viral load and kill infected cells by recruiting and activating effector immune cells. Moreover, they can be long-acting and can collaborate with the immune system improving different mechanisms that can control HIV-1 infection. Considering that these mechanism depend on specific regions within the antibody molecule, these regions can be modified to improve the global activity, making antibodies more potent or with a better *in vivo* pharmacokinetic profile. However, the contribution of antibodies to the removal of the HIV reservoir is still poorly defined.

### Isolation of bNAbs from HIV-infected individuals: selecting the best candidates

Probably the major limitation of antibodies in their continuous race against HIV is the structural complexity and the unprecedented variability of the HIV envelope glycoprotein (41). Although the humoral response against HIV Env is strong, most of anti-HIV Env antibodies fail to bind to complex trimeric epitopes and those recognizing the trimer usually are strain specific. Only a small proportion of anti-Env antibodies are bNAbs, i.e., block a large number of Env variants (58).

The isolation of bNAbs to inform on vaccine design and protective mechanisms has been a challenge over the last years. Initial efforts identified the anti-CD4bs antibody IgGb12, the anti-glycan 2G12 and the anti-MPER antibodies 2F5 and 4E10 as neutralizing antibodies. However, their potency was low (in the microgram/ml range) and the breadth limited (59, 60). New molecular and cellular screening technologies (61, 62) and large efforts by several laboratories have extremely helped to identify newer, broader, and more potent antibodies (40). Current bNAbs target vulnerability sites of HIV Env, show antiviral activity in the ng/ml range and cover a wide range of HIV isolates from different clades. In general, anti-CD4bs antibodies, such as VRC01, 3BNC117, VRC07, or N6 show intermediate potency and high breadth (>80% of a panel of 200 isolates), being the N6 antibody almost panneutralizing (63–66). In contrast, antibodies directed against the V2 loop, such as PG9 or PG16 (67), or against the V3 loop, such as 10-1074 or PGT121 (68) show higher potency but lower coverage, being able to neutralize roughly a 60% of circulating HIV isolates. The case of the anti-MPER antibody 10E8 is also relevant, it shows the widest coverage but has a limited potency (65).

The excellent combination of potency and coverage of recently isolated bNAbs have allowed for their clinical development. Over the last years, VRC01, 10-1074, 3BNC117, VRC07 or their combinations have reached clinical trials in humans (69–72), opening the door for antibody-based therapies to treat and cure HIV infection (40).

### Engineered antibodies

Despite the remarkable potency of bNAbs isolated from HIV-infected individuals, they show several limitations to become referent drugs in HIV treatment. Firstly, the number of antibodies is still low and there is a need for more potent antibodies particularly for some specificities, such as the MPER or the new vulnerability sites. Secondly, the number of isolates neutralized by some bNAbs is still low, and therefore combination strategies should be envisaged, increasing the development and production costs. For this reason, a wide range of modifications have been made to naturally occurring antibodies in order to increase their potency, coverage or pharmacodynamics behavior (40). The technologies to modify antibodies have grown as immunotherapies against several human diseases, such as cancer or autoimmune diseases emerged (73). Current technologies encompass modifications in the variable or the Fc regions of antibodies to modulate their antigen binding and their effector functions, respectively.

Several of those modifications have been applied to anti-HIV bNAbs. Huang et al. designed an asymmetric bispecific antibody containing the variable regions of an anti-CD4bs antibody and the 10E8 anti-MPER bNAb. This construction showed almost panneutralizing activity with a median IC50 of 2 ng/ml (74). Similarly, Bournazos et al. designed a flexible bispecific IgG3 antibody containing the 3BNC117 and the PGT135 variable regions (75). Other strategies involve the addition of specificities at the C-terminal end of the IgG. In this regard, Gardner et al. added a peptide that binds to the coreceptor binding site at the end of a CD4-IgG1 fusion protein resulting in the molecule eCD4-Ig that show also panneutralizing activity with a median IC50 in the ng/ml range (76) and maintain ADCC activity (77). Further modifications in antibody specificity involve the addition of a second variable region to the antibody arms, if this strategy is combined with an asymmetric chain assembly the result is a trispecific antibody. Such a molecule has been recently designed by combining the variable regions of the VRC01, the PGDM1400, and the 10E8 antibodies yielding a highly active and virtually panneutralizing molecule (78). Finally, increased neutralizing activity can be also achieved by introducing small modifications in the variable regions of antibodies; this is the case of antibody VRC07-523, an antibody with improved profile designed by computational bioinformatics, and structure-guided design (64), or the antibody NIH45-46^G54W^ that shows improved neutralization than its wild type counterpart (79). All above described engineered antibodies have been tested in animal models with excellent prophylactic results.

Modifications to the Fc moiety of antibodies have been also implemented into anti-HIV bNAbs. It is well known that glycosylation of the Asn297 residue in IgG1 is required for binding to CD16 and ADCC activity, and that removal of a fucose residue in the sugar chain increases binding to CD16 and ADCC activity of 2G12 and PG9 antibodies (80, 81). These modifications have been also applied to newly designed multivalent antibodies to improve ADCC-mediated removal of HIV-infected cells (82). However, the most common modification of the Fc is related to the binding to FcRn. Increasing the affinity of IgG for FcRn at pH 6 reduces lysosomal degradation of IgG by endothelial cells and extends IgG plasma half-life. For instance, the M428L and the N434S mutations in Fc, conferring an improved binding profile to the FcRn, have been introduced into the VRC01, the 10-1074 and the 3BNC117 antibodies, resulting in increased *in vivo* plasma half-life (83) and also reaching a higher concentration at mucosal level (56).

Alternatively, the Fc portion of antibodies can be completely removed. The resulting small molecules called dual-affinity re-targeting proteins (DART) are composed of two antigen-binding variable regions linked by a short sequence allowing the recognition of two different antigens (84). When one specificity is directed against the HIV Env and the other against CD16 or CD3, the resulting molecules may serve as a linker between HIV-infected cells and effector cells, NK or CTL, respectively (40). These molecules, despite their short plasma half-life (85, 86) may facilitate the removal of HIV-infected cells, exploiting the full repertoire of CD8+ T cells, as antigen specificity is not provided by the effector cell, but by the molecule.

## Antibodies in HIV therapy and the control of HIV reservoir

Immunotherapies using hyperimmune plasma were assessed in HIV-infected individuals before cART development in early years of HIV pandemics with disappointing results (87). Some years later, the available recombinant antibodies at that moment, the anti-glycan 2G12 and the anti-MPER 2F5 and 4E10 mAbs, reached clinical trials. All antibodies were safe in HIV-infected individuals (88, 89), although clinical effect was limited as shown by Trkola et al (90). These authors used a combination of the above-mentioned antibodies to treat HIV-infected individuals undergoing interruption of cART (Table 1). The effect on viral rebound was minimal, only observed in two of eight individuals and evidenced a partial activity of the antibody 2G12 (90). Despite the lack of clinical benefit, these trials demonstrated that treatment with anti-HIV antibodies was safe and paved the way for future treatments with newer and more potent antibodies.

**Table 1:** Latest Human Clinical Trials Involving bNAbs.

**Antibody(ies)**	**Target population**	**Endpoint**	**Dose^a^**	**References**
2G12 + 2F5 + 4E10	HIV infected cART interruption	VL rebound	30 mg/Kg IV	(84)
VRC01	HIV infected Untreated	VL decay	Up to 40 mg/Kg IV/SC	(68)
3BNC117	HIV infected Untreated	VL decay	Up to 30 mg/Kg IV	(69)
10–1074	HIV infected Untreated	VL decay	Up to 30 mg/Kg IV	(70)
VRC01LS	HIV uninfected	Safety	Up to 40 mg/Kg IV/SC	(91)
VRC07-532LS	HIV uninfected	Safety	Up to 40 mg/Kg IV/SC	(92)
10-1074 + 3BNC117	HIV uninfected untreated	Safety	Up to 30 mg/Kg IV	(93)

a *IV, intravenous; SC, subcutaneous*.

### NHP models

The isolation and development of bNAbs has mostly focused on the prophylactic activity, generating a plethora of work that demonstrates the efficacy of antibodies delivered as passive infusions or though gene therapy to protect animals from HIV acquisition, excellently reviewed in Pegu et al. (94). The first clear evidence of a therapeutic activity of antibodies came from humanized mice models (95). Klein et al. infected immunodeficient mice humanized with human CD34+ cells with the HIV isolate YU-2. Three weeks after infection animals were treated with monotherapy with the antibodies 3BC176, PG16, NIH45-46^G54W^, PGT128, or 10–1074. The effect of monotherapy on viral load (VL) was minimal, while the combination of three antibodies had some effect and the combination of all five antibodies achieved a sustained reduction of VL. Thus, combinations of potent monoclonal antibodies could effectively control HIV-1 replication at least in mouse models. This observation was relevant as the efficacy of antibodies against cell-to-cell HIV transmission, one of the most efficient *in vivo* mechanisms of HIV spread, was under discussion at that time (91, 96–98).

Further evidence of therapeutic activity of antibodies came from two different experiments in SHIV-infected Rhesus macaques (63, 92). Barouch et al. demonstrated that PGT121 administration to SHIV SF162P3-infected Rhesus resulted in a rapid decline of viral replication that was dependent on the level of VL at baseline, and was sustained for those animals with lower VL (92). Similarly, Shingai et al. treated SHIV AD8-infected rhesus macaques with the anti CD4bs antibody 3BNC117 or the anti-V3 antibody 10-1074 either as monotherapy or in combination. In monotherapy, the 10-1074 antibody caused a transient (4–7 days) undetectability of VL followed by virus rebound and appearance of mutations in the virus that confer resistance to the antibody. When administered together, 3BNC117 and 10–1074 induced a 3–5 weeks sustained suppression of VL without evidence of emergence of resistant viruses (63).

Despite these promising data, the effect of bNAbs on the HIV reservoir is still elusive. Initial work on humanized mice described an impact of bNAbs on HIV-infected cells by a mechanism related to their Fc effector functions (53, 93, 99). However, the most exciting results suggesting an effect of antibodies on the HIV reservoir have been recently generated in two papers demonstrating a clear long-term effect of antibody treatment in NHP (57, 100). Borducchi et al. treated SHIV SF162P3-infected rhesus 1 week after SHIV exposure with serial doses of PG121 antibody, the LRA GS-9620 (a TLR7 agonist) or a combination of both. Treatment was concomitant to cART, and after immunotherapy, cART was maintained for more than 2 years. After cART interruption all control animal showed rapid rebound of VL. TLR7 agonist monotherapy had a modest effect on viral rebound, while antibody treatment and particularly the combination of antibody and TLR7 agonist delayed or prevented VL rebound in most animals. Interestingly, all animals treated with combination therapy showed spontaneous control of viremia after cART cessation (100). Similarly, Nishimura et al treated SHIVAD8-EO infected Rhesus with a combination of 3BNC117 and 10-1074 bNAbs during acute infection. The effect of the treatment was a long-lasting control of HIV replication in roughly half of animals (six of thirteen) that seems to be related to an enhanced CTL mediated control of viral replication (57), suggesting a not yet defined immunomodulatory effect of antibodies in the course of HIV infection, probably related to the enhancement of immune responses by ICs captured by DCs reported in other viral infections (48) Although, these experimental designs are hard to replicate in humans due to the very early therapy schedule, these results show for the first time the potential of bNAb immunotherapy in HIV cure (either sterilizing or functional).

### Human trials

The progresses in bNAb-based therapy in humans are slow due to regulatory constraints. However, besides early trials using first-generation antibodies (90), several second-generation bNAbs have reached human clinical trials in passive administration assays. A summary of the most recent trials is shown in Table 1. The anti-CD4bs antibodies VRC01, VRC07-532LS, and 3BNC117 and the anti-V3 loop antibody 10-1074 were tested in HIV uninfected individuals demonstrating to be safe and well tolerated (101). Pharmacokinetics was variable among the different antibodies tested, showing a plasma half-life close to 15 days, as expected for a therapeutic IgG. Interestingly, the VRC01LS antibody, a derivative of VRC01 containing the M428L and the N434S mutations in the Fc showed increase plasma half-life (71 ± 18 days) and was also safe in a Phase I trial (102). Similar results have been reported for the VRC07-532LS antibody containing a similar set of mutations in the Fc and showing also an extended half-life of 33 ± 18 days (103). Furthermore, combination of 3BNC117 and 10–1074 does not seem to alter their individual pharmacokinetics or safety profile (104).

VRC01, 3BNC117 and 10-1074 antibodies have been also tested in untreated HIV-infected individuals (69–71). The behavior of all antibodies was similar, showing the weaknesses and strengths of antibody monotherapy. Treatment led to a reduction of VL in most of individuals (ranging from a 1.1 to 1.8 logs) with more sustained suppression in individuals with lower baseline viremia. However, in some patients the antibody had no effect on VL, due to the presence of viral variants that were insensitive to neutralization activity (resistant viruses). Interestingly, most individuals that transiently reduced VL showed in the rebounded virus mutations conferring reduced sensitivity to the therapeutic antibody, suggesting rapid development of HIV resistance (69–71). Furthermore, due to active antigen removal, the plasma half-life of antibodies was shorter than in HIV uninfected individuals (69–71). In addition to these expected data, antibody treatment also revealed further relevant information. The passively administered antibody 3BNC177 altered the kinetics of HIV-1 suppression in infected individuals, suggesting an active effect of the antibody on infected cell clearance. Consistent with data from animal models, the mechanism would require Fcγ receptor engagement (105). Moreover, this antibody also seems to significantly improve neutralizing responses to tier 2 viruses in most study participants, suggesting beneficial immunomodulatory properties as described in other animal models (48). In contrast, treatment with VRC01 antibody had no impact on the size of HIV reservoirs in cART treated HIV-infected individuals at least 4 weeks after two antibody infusions (69).

## Antibodies in prevention strategies: targeting sexual and vertical transmission

Besides the potential role in cure intervention, bNAbs may also be useful in preventative strategies further contributing to control and eradicate the HIV pandemic. Complementing the therapeutic use of antibodies, human trials have been started to determine the efficacy of the antibody VRC01 in protecting HIV uninfected individuals at risk of HIV acquisition. A large study in men who have sex with men (MSM) in US and women in Africa (the AMP study) is currently conducted (106, 107) to test antibody-mediated protection of sexual HIV transmission. However, one of the most attractive fields for the prophylactic use of antibodies is mother to child transmission (MTCT), excellently reviewed in (108).

The evolution of the prophylactic use of antibodies has paralleled the therapeutic application. Pioneer work involved purified immunoglobulins from HIV serum to block MTCT by treating HIV-1-infected pregnant women showing safety but a complete lack of efficacy (109, 110). Additional studies confirmed these early result (111). Again, the isolation of second-generation bNAbs, along with the recent data obtained in NHP and humans, has renewed the interest on the use of nNAbs to block MTCT. Although there is profuse information on the excellent prophylactic activity of bNAbs in adult uninfected rhesus macaques challenged with HIV (94), specific studies on animal models of MTCT are more recent. Hessell et al. inoculated orally 1-month-old rhesus macaques with SHIV and treated them subcutaneously with VRC07 and PGT121 1 day after virus exposure. All treated animals showed no SHIV or anti-SHIV T cell responses in blood or tissues at necropsy, and importantly no virus emerged after CD8 T cell depletion (112), suggesting that early passive immunotherapy can eliminate early viral foci and thereby prevent the establishment of viral reservoirs in newborns.

These preclinical data have fostered the clinical use of bNAbs in prevention of MTCT in the hallmark the International Maternal/Adolescent AIDS Clinical Trials Network (IMPAACT). Indeed, the P1112 study is currently ongoing and evaluates the safety and PK analysis of the antibody VRC01 in HIV-exposed newborns and will also include the VRC01-LS in one of the arms (113). Moreover, the 2008 study is also being conducted; this is Phase I/II study that uses VRC01 in combination with cART to analyze the effect on the clearance of HIV-1-infected cells in infants. The data from both studies will inform on potential benefits of antibodies to reduce MTCT and the possibility to expand bNAb-based eradication strategies to infants.

## Future clinical perspectives and remarks

Preliminary data on the therapeutic use of bNAbs isolated from HIV-infected individuals suggests a strong potential in HIV cure strategies. However, their clinical use is limited by the availability of antibodies, the antiviral potency and the preexisting resistances. To overcome these limitations, a large array of synthetic multifunctional antibodies with higher potency, breadth of neutralization and tailored effector functions (including CTL mediated killing) are under development to feed the pipeline of antibody-based therapies and to improve our current arsenal of anti-HIV drugs. It should be noted that those newly designed antibodies show a potency equivalent to the most active antiretroviral (IC_50_ in the pM range), offer minimal natural resistance (and given their multifunctional mechanism of action an anticipated high genetic barrier) and a wide range of immunological effect, most of them not yet completely understood.

The clinical setting for antibody treatment is also an open field. A reduction of the HIV reservor by passive antibody administration to chronic HIV-infected individuals would be desirable, but could be incomplete. Despite preliminary promising data, bNAbs will probably require combination with LRAs or therapeutic vaccines to eradicate HIV. Early treatment is probably a more promising scenario. Replicating excellent data obtained by early treatment of NHP in humans would speed the implementation of this type of therapies. Although implementation in adults is complex due to the challenging detection of early infection, a promising future is envisaged in MTCT settings. New technologies will also help bNAb therapy. Passive administration can be replaced by gene therapy using Adeno-Associated Viruses (AAV), a strategy that has provided excellent results in NHP and that is currently being tested in humans (76, 114–116).

## Author contributions

JB and JC drafted the manuscript. JC and BC reviewed the manuscript and made substantial, direct, and intellectual contributions to the work. All authors approved it for publication.

### Conflict of interest statement

JC, BC, and JB are founders of AlbaJuna Therapeutics, SL. JB and BC received grants from MSD unrelated to this work.
